# Overview of Power Electronic Switches: A Summary of the Past, State-of-the-Art and Illumination of the Future

**DOI:** 10.3390/mi11121116

**Published:** 2020-12-16

**Authors:** Immanuel N. Jiya, Rupert Gouws

**Affiliations:** 1 Department of Engineering Sciences, University of Agder, 4879 Grimstad, Norway; 2 School of Electrical, Electronic and Computer Engineering, North-West University, Potchefstroom 2520, South Africa; rupert.gouws@nwu.ac.za

**Keywords:** bipolar transistors, insulated gate bipolar transistors, power semiconductor devices, power transistors, power semiconductor switches, thyristors

## Abstract

As the need for green and effective utilization of energy continues to grow, the advancements in the energy and power electronics industry are constantly driven by this need, as both industries are intertwined for obvious reasons. The developments in the power electronics industry has over the years hinged on the progress of the semiconductor device industry. The semiconductor device industry could be said to be on the edge of a turn into a new era, a paradigm shift from the conventional silicon devices to the wide band gap semiconductor technologies. While a lot of work is being done in research and manufacturing sectors, it is important to look back at the past, evaluate the current progress and look at the prospects of the future of this industry. This paper is unique at this time because it seeks to give a good summary of the past, the state-of-the-art, and highlight the opportunities for future improvements. A more or less ‘forgotten’ power electronic switch, the four-quadrant switch, is highlighted as an opportunity waiting to be exploited as this switch presents a potential for achieving an ideal switch. Figures of merit for comparing semiconductor materials and devices are also presented in this review.

## 1. Introduction

In spite of the decreasing world population growth rate over the years, the human population on planet earth has continued to grow reaching just under eight (8) billion people so far [1,2,3]. This growing population coupled with a drastic increase in industrialization on a massive scale has brought about huge demands for energy. The international energy agency (IEA) projects a 25% increase in the global primary energy demand between the years 2017 and 2040. It is also predicted that if no further improvements occur in the energy efficiency of the world, it could lead to a 50% increase in energy demands [4].

This rather radical increase in energy demand alongside the damaging effects of climate change and degradation of planet earth has resulted in an aggressive exploitation of non-renewable and pollutant sources of energy over the years. To combat the detrimental effects of man’s long-standing pollution of the environment and attempt to attain a state of balance and environmental purity on planet earth, there has been a rise in the utilization of renewable energy sources. Although the supply of energy from renewable energy sources to the energy supply mix continues to grow, in order to reach this state of balance and purity in the energy cycle, there needs to be a consistent increase in the efficiency of energy generation, supply and utilization. This need defines the drive of the power, energy and electronics industry and thus the drive of the power electronics industry [5,6,7].

The progress of the power electronics industry has been contingent on the level of progress in the semiconductor power device industry, otherwise known as the power electronic device industry and this particularly on the advancements in power electronic switches [8]. The power electronic switch industry has grown and improved drastically over the years and is now in a transitional phase from the more common silicon semiconductor technologies to a totally new phase of wide band gap (WBG) technologies [9,10,11,12,13,14,15,16]. This new phase is characterized by an improvement in the various characteristics and manufacturing techniques already in place for power electronic switches [17,18,19,20,21,22,23,24,25,26]. Generally speaking, advancements in power electronic switches are primarily to increase the switching speed, that is to reduce the time it takes for the switch to turn-on and turn-off, and to increase the power the switch is capable of handling [27,28].

This review article seeks to present an overview of a rapidly expanding industry and highlight the key opportunities for growth and improvements that exists. This is done to bring new stakeholders in the field (students and researchers) up to speed and to draw the attention of research, development, and manufacturing stakeholders to the opportunities available to be exploited in order to foster more rapid growth in the industry. This review article is unique in that it illuminates the fully controllable bi-directional switch which could potentially revolutionize the power electronics device industry in such a way that it brings the industry to a better footing in this race towards improving energy efficiency [6,7].

## 2. Brief History

The mercury arc rectifier (MAR), developed in the year 1900 is reported to be the first power electronic device to have been developed [29]. This was quickly followed by the development of other power electronic devices like the phantron, thyratron, metal tank rectifier, grid controlled vacuum tube rectifier, magnetic amplifier and ignitron [29]. The power electronics industry, however, did not really take off until the thyristors or silicon-controlled rectifiers (SCRs) came into play. The SCRs were proposed by Bell laboratories and General Electric (GE) produced them commercially in the early 1950s [30]. This robust SCR quickly got adopted in rectifier and cycloconverter circuits, thus replacing the MARs. However, the inability of the SCR to be turned off directly from its gates proved to be quite a hurdle, thus slowing down its adoption for other applications beyond the line-commuted mode of action. Several techniques to aid the turn-off of SCRs were proposed but the need for a device which could be turned off directly at the gate became very important as the techniques proposed to aid the turn-off of SCRs proved impractical at high powers, thereby limiting its application [31]. This need birthed the discovery of transistors [32,33].

In November 1947, Walter Brattain and John Bardeen discovered operation of transistors at Bell Labs [34,35,36]. Vacuum tubes were able to operate at low frequencies and thus, for operation at higher frequencies, the concept of point contact invented for solid-state rectifiers was used in transistors [34,37]. Research in the field of quantum mechanics clarified the concepts of electron conductivity and structure metals, semiconductors, and insulators. Other solid-state devices were being invented by different researchers, which include Field effect transistors (FETs) in 1926 by Lilienfeld [38], a solid amplifier with three metal leads by Pohl and Hilsch in 1938, and n or p-type single silicon crystals by Russell Ohl in 1940 [39]. Development of radio detection and ranging (RADAR) systems required shorter wavelength or high frequency which improved research in low capacitance solid state devices considering silicon and germanium [40,41]. The very first transistor had the base of germanium crystal and two electrode tips with the first coating layer of metal acting as collector, then wax and then again metal, acting as the emitter [42]. The electrolyte was connected to the emitter and the current flowing from emitter to collector was modulated by the base current. Later this transistor was improved in design and Shockley introduced the concept of minority carriers with bipolar junction transistor theory [40,43,44]. In April 1950, with very thin germanium crystal as the base, first negative-positive-negative (NPN) transistor was created [45]. Later, using diffusion technique, first germanium NPN transistor working at 500 MHz and then silicon NPN transistor working at 120 MHz was developed [39]. Zone refining technique was discovered to get highly pure semiconductor crystals, by moving hot zone along the tube which accumulates melted semiconductor material with impurities. After this, silicon was preferred for transistors, due to its property of having large band gap at low temperatures unlike germanium [46]. By doping a semiconductor material with impurities of antimony or arsenic, n-type silicon and by adding gallium or boron, p-type silicon was created. And by forming a transition region and cut bars acting as diodes, PN junctions were formed. Out of the two methods developed for a narrow base layer, rate-growing process, and diffusion, diffusion process was able to produce more controllable and narrow base layers and hence was preferred [40].

Bipolar junction transistor (BJT) is current operated semiconductor device and thus has applications in current amplification, whereas vacuum tubes were being used as voltage amplifiers. The first integrated circuit was prepared using two BJTs on a single chip in 1958 [47], since implementing complicated circuits were easy with BJTs. Later, due to advantages of metal oxide semiconductor field effect transistor (MOSFET) technology including simpler IC processing and packing more devices on the single chip, ICs were produced using MOSFETs since the 1970s [32]. The concept of insulated gate field-effect transistor (IGFET) insulated gate field-effect transistor, similar to vacuum in vacuum tubes is been used in MOSFETs. The insulated gate produces a traverse electric field to modulate the current in semiconductor channel. BJT had surface problems affecting leakage current and device stability, few of which were addressed by the work of M. M. Atalla in 1958, by thermally grown silicon dioxide [48]. P-channel MOSFET device using silicon surface passivation technique, proposed by Atalla in 1960, was first implemented by Fairchild Semiconductor Corp. to improve planar diffusion fabrication of BJT [32]. In 1964, RCA and Fairchild both came up with first commercial discrete MOS transistors [49]. The need of miniature ICs with then developed monolithic IC technology increased the importance of MOSFET technology [50]. Advantages of MOSFET over BJTs made them preferred technology for computer applications which include few processing steps implying more fabrication products with less cost. Unlike BJTs, scaling down without performance changes; and decreased power dissipation and higher speed with reduced signal delay [51]. From 1970, the first single-chip 1024-bit MOS IC memory, and in 1971, first microprocessor 4004, till 1990, 80,486 microprocessor chip, by Intel, MOS technology became inevitable [32].

The development of the MOSFET is reputed to be the very first successful marriage of discrete power semiconductor manufacturing technologies and the modern integrated circuit [46]. Although the MOSFET received wide adoption, it had a few limitations, because it is a minority carrier device, its ON-state voltage is dependent on the ON-state resistance of the drain to source, thus leading to the non-viability of MOSFETs for high voltage applications. On the other hand, there was a continuous research into the SCR to improve its turn-off capability by reducing its turn-on gain [52]. These researches gave birth to the gate-turn-off thyristor (GTO) [53,54]. However; the GTO requires a high turn-off control current through the gate, this coupled with its relatively higher cost limited its application to only inverters with relatively low KVA ratings of a few hundreds [55].

The insulated-gate bipolar transistor (IGBT) was developed to overcome the drawbacks of bipolar transistor of low current gain, by combination of MOSFET and bipolar transistor [37]. Thus, IGBT has applications in medium and high-power applications. From 1970 to 1980, researchers were working on combination of MOSFET and bipolar transistor to improve the poor latch-up condition of these devices [32]. Finally in 1985, Nakagawa successfully designed non-latch up IGBT structure with N^+^ type surface and shallow impurity P^+^ regions with high impurity [56]. It overcame the disadvantages of insulated gate rectifier (IGR), of slow switching speed, short circuit safe operating area (SOA) and reverse biased SOA. To improve the switching characteristics of IGBT, Goodmann introduced n^+^ buffer layer in 1983 [57], with the trade-off of forward voltage drop. Since IGBT has power MOSFET structure, to improve forward drop voltage, trench-gate technology was implemented with ultra large scale integration (ULSI) techniques [58]. Close arrangement of trench cells causes the big channel width problems of short circuit, which only can be solved using current limitation circuits. The combination of injection enhancement (IE) and IGBT formed a new structure called as IEGT with characteristics of low forward voltage drop, improved surface gate structure, and carrier enhancement. Takahashi further introduced improvement in IEGT which is known as CSTBT (carrier store trench gate bipolar transistor) by adding n-doped layer below p-type trench cell [37].

**Figure 1:**
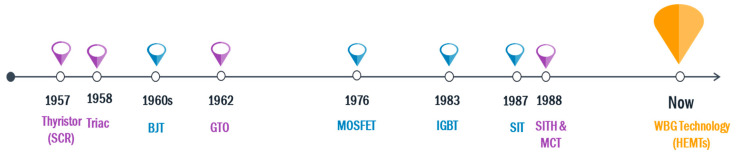
Time line of the introduction of modern power electronic switches.

In Figure 1, a timeline of the semiconductor devices’ introduction is presented. Currently, the future of the semiconductor technology industry is looking into the utilization of semiconductor materials such as silicon-carbide (SiC), gallium nitride (GaN) and aluminum nitride (AlN) which have energy band gap of about 2 eV, 3.4 eV and 6.2 eV respectively, for manufacturing of power electronic devices [59,60,61,62,63,64,65,66]. These semiconductors have extremely high mechanical, chemical and thermal stability [67,68]. These impressively high stability characteristics, alongside the wide breakdown fields and very low ON-state resistances (it is reported that the R_DS(ON)_ could be as low as 20 times that of silicon devices) of the devices made from wide band gap semiconductors makes this technology very promising and hence the potential future of power electronics [69]. In addition to these advantages, wide band gap (WBG) devices are capable of operating at high frequencies thus having the potential to require much lower filter sizes when they are utilized in converter circuits [70,71,72,73,74,75,76,77,78,79,80]. The efficiency and power density of the converter circuits is also positively impacted when WBG switches are used. For now WBG technology is used mainly in diodes, MOSFETs and IGBTs [81,82,83,84,85,86,87]. There is, however, no reasonable doubt that WBG technology is the key to the future of power electronic switches [88,89,90].

## 3. Classification of Power Electronic Switches

**Figure 2:**
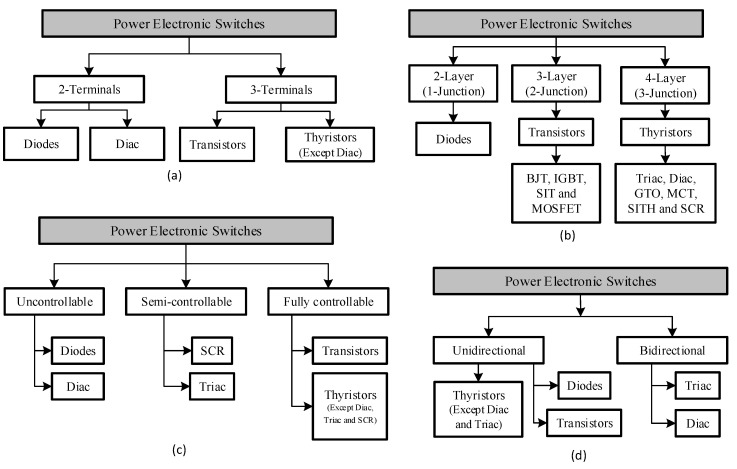
Classification of modern power electronic switches based on (**a**) number of terminals, (**b**) number of layers or junctions, (**c**) controllability and (**d**) bidirectional capability.

Modern power electronic devices can be classified in a number of ways based on the number of terminals, the number of pn junctions, level of controllability, bidirectional capability, and the gate signal requirements to name just a few. In Figure 2, the more prominent classification of the modern power electronic devices is presented.

Taking the number of terminals, power electronic devices are broadly divided into two groups, the 2-terminal and the 3-terminal devices. The two terminal devices are devices whose switch state solely depends on the circuits which they are externally connected to. The diode and the diac are prime examples and occupants of the 2-terminal device category. As it can be observed in Figure 2a, the 3-terminal device consists of the thyristor and the transistor. These are devices whose switching state does not solely depend on the external circuit to which they are connected but also depends on the driving signal at their respective gates.

In Figure 2b, the classification of modern power electronic switches based on their number of junctions or layers is presented. This classification could be one of the most appropriate and robust classification of semiconductor switching devices. Based on this classification, there are three categories, namely: the 1-juntion or 2-layer, the 2-juntion or 3-layer and the last one which is a 3-junction or 4-layer group of power electronic switches.

The 1-juntion or 2-layer group consists of the diode, the 2-junction or 3-layer group consists of the transistor and the 3-junction or 4-layer group consists of the thyristor families of power electronic devices. This classification was earlier referred to as the most robust because all of the different families find themselves in their respective family-groups and it speaks more to their structure than to just their characteristics.

A third classification of the power electronic switches is based on their controllability. In this classification, there are three groups, the uncontrollable switches, semi-controllable switches, and the fully controllable switches. The uncontrollable switches consist of the diode and the diac just like in the 2-terminal classification based on their number of terminals. These switches are in this class because the turn-on and turn-off cannot be controlled and is solely determined by the external power circuit. The semi-controllable switches can be turned on but cannot be turned off directly from the gate. In this category, the thyristor, specifically the silicon-controlled rectifier (SCR) and the triac are prime members. As can be seen in Figure 2c, the last group, the BJT, MOSFET, GTO, static induction thyristor (SITH), static induction transistor (SIT), IGBT and MOS-controlled thyristor (MCT) are all members.

The fourth main classification is based on their bidirectional or unidirectional current capability; this is presented in Figure 2d. It is seen that only the triac, diac, and the RCT are capable of bidirectional current capability while the rest are only capable of unidirectional current flow. It is worthy of note that most commercially available MOSFETs have body drain diodes which are antiparallel to the MOSFET switch hence giving it a bidirectional capability but the flow of current through the body diode cannot be controlled externally.

Other classifications of power electronic switches include the gate signal requirement and the type of charge carriers utilized in the switching technology. For the gate signal requirements, some switches such as BJTs, MOSFETs, IGBTs, and SITs require a continuous gate signal to keep the switch operational, the GTO, MCT and the SCR only require a pulse gate signal while the diode does not have a gate to start with. For the charge carriers used, this classification is in two groups, the majority and the minority charge carriers. In majority carriers, only one type of charge carrier is used in the transportation of current through the semiconductor. That is, either electrons or holes in an n-type semiconductor or p-type semiconductor respectively. Examples of switches that utilize majority carriers are Schottky diodes and MOSFETs. On the other hand, minority carriers use both electrons and holes to transport current through the semiconductor material. Examples of switches utilizing minority carriers are thyristors, BJTs and IGBTs.

### 3.1. Diodes

**Figure 3:**
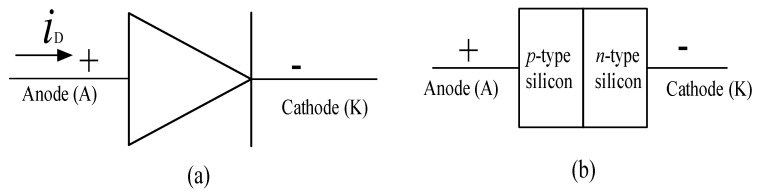
(**a**) Schematic symbol and (**b**) basic structure of a diode [91].

Diodes are formed from a pn junction of semiconductor material, as shown in Figure 3, they have just two terminals, the anode and the cathode [91]. A diode is an uncontrollable and unidirectional switch hence, its operation is determined by the direction of the flow of current in a circuit [92].

A diode is basically a short circuit when it is forward biased, that is when current is flowing from the anode to the cathode and conversely it is an open circuit when it is reverse biased, that is when the direction of the flow of current is in the opposite direction in the circuit. There are generally three types of diodes, the power diodes, schottky diode and the zener diode [93].

The power diode can be further classified into two based on their characteristics and applications. The two classes include the line-frequency diodes and the fast recovery diodes. The line frequency diodes, also known as the general-purpose diodes, are used in line frequency applications (that is frequencies around 50–60 Hz) such as in AC rectification. They are characterized by slow recovery times and can be used in applications requiring high voltage and high currents (>5 kV and kA respectively) [94].

The fast recovery diodes as their name implies are characterized by fast recovery times and are therefore utilized in high applications such as traction systems, induction heating and uninterruptible power systems (UPS). The downside with fast recovery diodes when compared to line frequency diodes is that they cannot operate at such high voltages and currents as line frequency diodes can [95].

**Figure 4:**
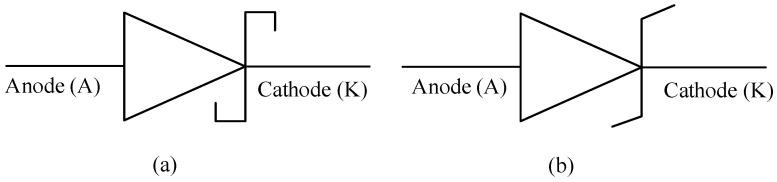
Schematic of (**a**) a schottky diode and (**b**) a zener diode [95].

The other types of diodes are the Schottky and Zener diodes, the schematic representation of these diodes are presented in Figure 4a,b respectively. Schottky diodes, named after Walter Schottky [96], instead of being formed from the regular pn semiconductor junction and rather formed from a metal-n semiconductor junction. The metals could be made of platinum or tungsten. This metal-n type semiconductor material junction affords the Schottky diode to have a low on-state voltage drop of as low as 0.1 V. Although its breakdown voltage is much lower than that of the power diode, it switches faster and has a high switching frequency but a higher reverse leakage current than the power diode. Due to these characteristics, Schottky diodes are often used in low voltage and high current applications like switched mode power supplies [97].

On the other hand, a Zener diode while still being made from pn semiconductor junction, in simple terms can allow flow of current when reverse biased as long as the voltage is above a specific value known as the Zener voltage, breakdown voltage or the Zener knee voltage. The Zener diode is used to produce a stable voltage (the Zener knee voltage) from a varying supply voltage [98]. Therefore, a Zener diode can be used to generate a reference voltage for an amplifier stage or as a voltage stabilizer in low current applications. The Zener diode must be implemented with a current limiting resistor in series with itself to keep the flow of current in the Zener diode at a safe level [94].

### 3.2. Transistors

**Figure 5:**
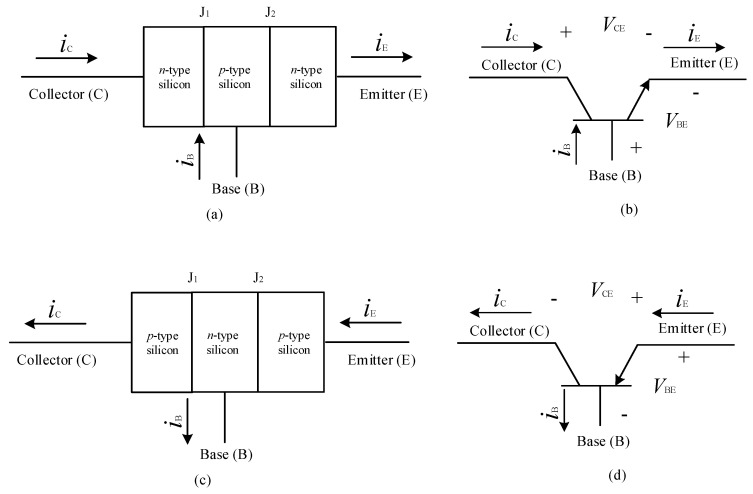
Structural diagram and schematic symbols of (**a**,**b**) an NPN transistor and (**c**,**d**) a PNP transistor [101].

When two PN junctions are combined together with the center layer of semi-conductor being common to the two outer layers, forms the transistor of either PNP or NPN type, the schematic representation and structure of BJTs is presented in Figure 5 [99]. Hence the name bipolar junction transistor (BJT), since it uses both electron and holes as charge carrier. In NPN transistor the current flows from collector to emitter, when the base is supplied with the positive voltage, while in PNP transistor the current direction is reverse i.e., from the emitter to collector when the base is supplied with a negative voltage [100]. Since, in NPN transistors, majority carriers are electrons and minority carriers are holes, the transistor is turned-on when electrons enter the base region; while in PNP transistor, majority and minority carriers are holes and electrons, respectively, thus the transistor is turned ON because of movement of holes into the base [101]. As mentioned earlier, combination of two PN-junctions, i.e., emitter-base junction and the collector-base junction with common semiconductor acting as the base, at the center, forms PNP or NPN type transistors, with the emitter, base and collector regions [102]. To achieve desired power gain, one junction is kept in forward biased while another junction is kept in reverse biased condition. The biasing type also determines the mode of operation in active mode, cut-off mode, and saturation mode. The polarity of bias junction is determined by the type of the transistor; consider PNP structure shown in Figure 5c,d [103]. For both type of transistors, emitter-base junction when in forward bias condition, due to drift, the majority carriers, either holes or electrons emitted from emitter enter the base region for recombination. Escaped remaining carriers get diffused in base region are carried forward into collector region by applying a reverse bias to the collector-base junction [104].

Because of high mobility of electrons, NPN transistors have high conductivity and thus, switching time in NPN transistors is faster than that of in PNP transistors. Thus, for switching and amplification applications, NPN transistors are preferred. Collector terminal in NPN and emitter terminal in PNP transistor are supplied with positive voltage [105]. For analog applications, polysilicon emitter BJTs are used. With new technology, the size of transistors is reduced which gives high current and high-frequency gain but also increase low-frequency noise [106]. In a study by [107], it was observed that, in NPN transistors, shot noise of the base current contributes to total noise, while in a PNP transistor, generation-recombination noise adds to the total noise.

**Figure 6:**
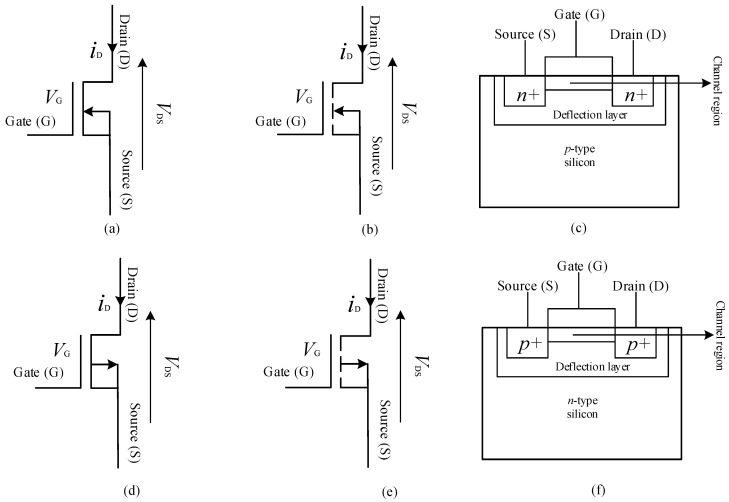
Diagrams showing (**a**) the schematic symbol of a depletion mode N-channel MOSFET, (**b**) the schematic symbol of an enhancement mode N-channel MOSFET (**c**) the structural diagram of an N-channel MOSFET (**d**) the schematic symbol of a depletion mode P-channel MOSFET, (**e**) the schematic symbol of an enhancement mode P-channel MOSFET and (**f**) the structural diagram of a P-channel MOSFET [110].

Metal oxide semiconductor field effect transistors (MOSFETs) are unipolar devices, since they have only one type of carrier, unlike BJTs. In MOSFETs, the current through the device is controlled by the voltage between its terminals, source, and gate [108]. In MOSFETs, the gate is insulated from channel using silicon dioxide (SiO_2_) material, labelled as the deflection layer in Figure 6 [109,110]. There are two kinds of MOSFETs, as shown in Figure 6, enhancement (E) mode and depletion (D) mode, enhanced MOSFETs (EMOSFETs) also known as insulated-gate field effect transistors (IGFETs) are widely used, because of polycrystalline silicon gate materials [111].

The structure of a depletion MOSFET (DMOSFET) is same as that of an enhancement MOSFET (EMOSFET), except in a DMOSFET, there is a narrow channel present between diffused source and drain into the substrate, adjacent to the insulated gate. N-channel and P-channel DMOSFET structures are same except for voltage polarities [112,113,114]. When an N-channel DMOSFET is operating in the depletion mode, the negative voltage on the gate repels the electrons from the channel. With an increase in gate voltage, at a certain point, the channel becomes completely depleted of electrons and channel current becomes zero. When an N-channel DMOSFET is operating in the enhancement mode, a positive voltage applied to the gate, attracts more electrons in the channel, increasing the channel conductivity [115].

EMOSFET structure does not include structural channel and only operates in enhancement mode. For an N-channel EMOSFET, when the applied gate to source voltage is positive, it attracts electrons and forms a narrow channel alongside SiO_2_ layer. With the increase in applied voltage; more electrons are attracted into the channel, increasing conductivity and below the threshold voltage, no channel is formed into the substrate [94]. Due to high drain to source resistance as a result of long thin lateral channel alongside insulation layer, EMOSFETs are suitable for low power applications. There are four types of EMOSFETs, the laterally diffused MOSFET (LDMOSFET), the V-groove MOSFET, the T-groove MOSFET, and the dual-gate MOSFET. The LDMOSFET structure has a shorter channel between the lightly doped source and drain [116]. It gives higher current and voltage than conventional EMOSFET and thus is suitable for high power applications. The VMOSFET structure has two source gates on top with a gate in between them and a drain at the bottom. The wide and short channel is induced between drain and source with less resistance, thus resulting in high current and power dissipation and better frequency response. The channel is formed along the V-groove between two sources and the drain, with thickness dependent upon the doping and diffusion time. In the TMOSFET structure, the source is all over top surface and drain is at the bottom, giving short vertical channel with more packing density than VMOSFET. Dual-gate MOSFETs have two gates giving low input capacitance and high-frequency applications with automatic gain control. To maintain the long-channel behavior of MOSFET, many parameters were altered during previous researches. In Brews et al., an empirical equation was proposed to find a relation between required parameters to be altered by observing long channel subthreshold behavior. The parameters considered were bias, doping level, junction depth and oxide thickness range [112,113].

**Figure 7:**
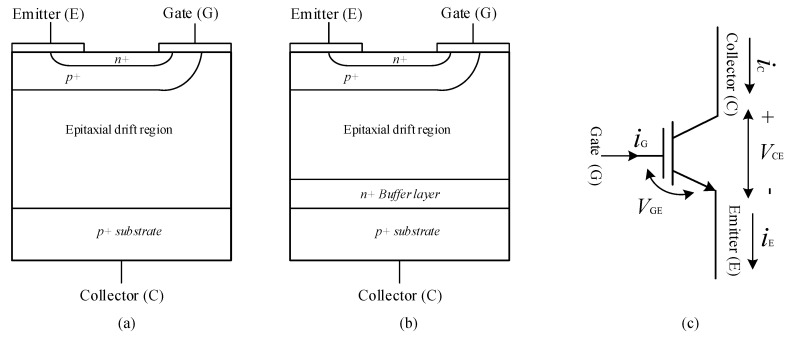
Diagrams showing the (**a**) the non-punch-through (NPT) structure of an IGBT (**b**) the punch-through (PT) structure of an IGBT and (**c**) the circuit schematic of an IGBT [117].

The insulated gate bipolar transistor (IGBT) combines advantages of the MOSFET and a BJT, this is since it has output characteristics of a BJT and voltage control like a MOSFET [117]. The circuit symbol of IGBT is as shown in Figure 7 alongside its structural diagrams. It has three terminals, gate from MOSFET, and an emitter and collector from BJT [94]. The insulated gate has no input current and thus, IGBT can be also called as voltage controlled BJT with faster switching characteristics [93].

During operation, a parasitic transistor *Q_p_* and parasitic resistor is formed within the IGBT device because of its NPNP-like structure [117]. These parasitic elements do not affect normal operation of IGBT unless under certain conditions, like if the collector current exceeds a certain level, then the parasitic transistor *Q_p_* may turn on. When *Q_p_* is turned on, combination of *Q_p_* and *Q*_1_ output transistor creates parasitic element. This parasitic element will keep the device in always ON mode, which is known as latch up condition. It can be avoided by controlling the device operation within specified limit values [118].

The IGBT structure is formed with NPNP-like four alternating semiconductor layers creating a thyristor-like structure. Thyristor operation is disabled using deep p^+^ diffusion and short circuit of P-base region using emitter [117]. There are two types of IGBT structures, the symmetric and asymmetric blocking structures [119]. Symmetric blocking IGBT structure is known as non-punch-through (NPT) IGBT device since because of lightly doped N-drift region, electric field does not spread across its entire width [120]. In asymmetric IGBT also known as punch through (PT) structure as shown in Figure 7, an N-buffer layer, also called as field stop layer is introduced in the N-drift region. Unlike NPT IGBT, electric field spreads across the entire width of the N-drift region. In PT IGBT, lower forward voltage drop can be achieved by reducing thickness of N-drift region. PT IGBT operates in the first quadrant of the v-i characteristics and thus is useful in DC applications [121].

The main differences between a NPT-IGBT and a PT-IGBT is that NPT-IGBTs are more thermally stable, have a higher carrier lifetime hence resulting in a lower forward voltage drop than PT-IGBTs. NPT-IGBTs are used in AC circuits while PT-IGBTs are utilized in DC circuits [122]. Also, the temperature coefficient of the on-state voltage of NPT-IGBTs is highly positive, making the NPT-IGBTs more well suited for device paralleling since the PT-IGBTs’ temperature coefficient of the on-state voltage usually tends almost to zero [123]. PT-IGBTs are capable of switching at a higher speed with even less energy consumed during switching when compared to NPT-IGBTs of the same voltage rating [124]. However, NPT-IGBTs are manufactured through a less expensive process (diffusion process technology) than the PT-IGBTs which are made in an N-epitaxial wafer profess.

**Figure 8:**
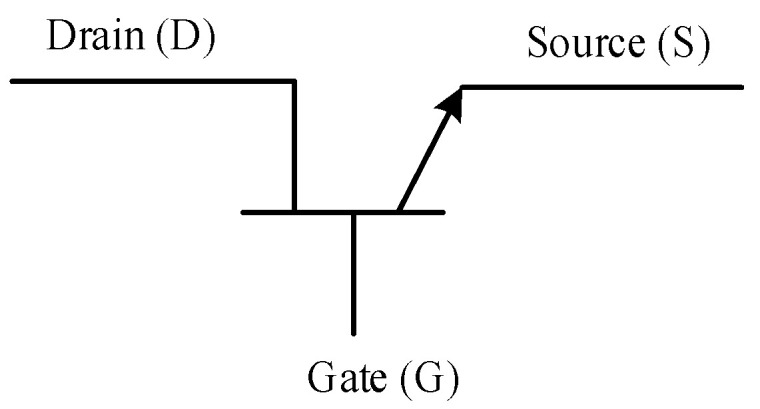
Schematic symbol of a static induction transistor [125].

Another prominent member of the transistor family is the static induction transistor (SIT), the schematic symbol of the SIT is presented in Figure 8 [125]. The SIT was introduced in 1987, just a year before the static induction thyristor by Tokin Corporation in Japan [126]. One of the main advantages of the SIT over a field effect transistor (FET), is that the SIT structure offers advantages in obtaining higher breakdown voltages, since is not limited by the surface breakdown between gate and drain, and therefore can operate at a very high current and voltage [127,128]. The SIT is a high frequency and high power device which is described to be basically a triode vacuum tube in solid state form [129]. It is used in induction heaters, high voltage low current power supplies, linear power amplifiers, ultrasonic generators, and AM/FM transmitters [130].

### 3.3. Thyristor Families

**Figure 9:**
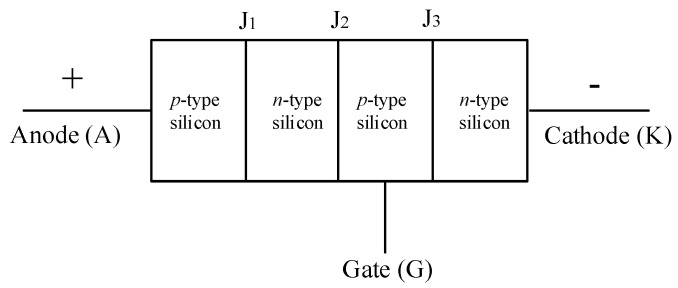
Basic structure of a thyristor [132].

The thyristor family of power electronic switches is as shown in Figure 9, a three terminal, four layer semiconductor device [131]. As can be seen in Figure 9, the four layers of a thyristor are made up from three alternating pn juntions. Some of the prominent members of the thyristor family includes: the SCR, GTO, static induction thyristor (SITH), MOS-controlled thyristor (MCT), triode AC switch (TRIAC) and the diode AC switch (DIAC) [132]. Thyristors are very often used to switch alternating currents and utilized when the power requirements (current and voltage) are relatively high (in excesses of a few thousand volts and amps) [133].

**Figure 10:**
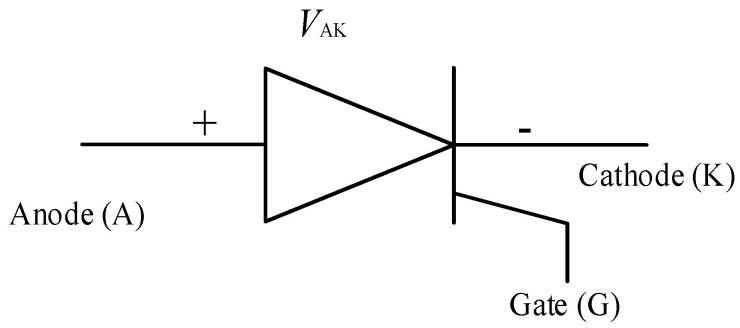
Schematic symbol of a silicon controlled rectifier (SCR) [132].

**Figure 11:**
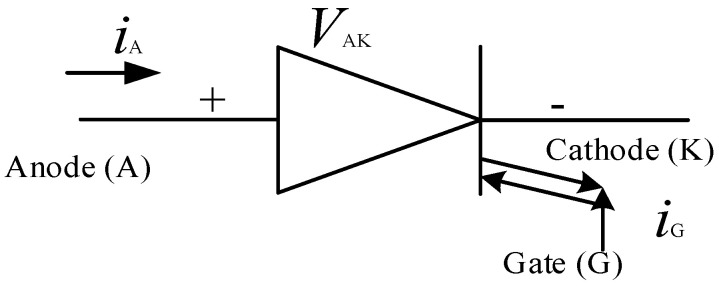
Schematic symbol of gate-turn-off thyristor (GTO) [134].

Figure 10 shows the schematic symbol of an SCR, which is also often referred to as a thyristor as well. An SCR is a unidirectional switch just like a diode but has a gate (G) which controls its turn ON. As it has been mentioned earlier, the turn-off of a thyristor is a quite complicated process and hence a new thyristor based switch called the gate turn-off thyristor (GTO) was introduced [134]. Although the thyristor quickly gained traction in many AC applications, such as in AC drives and uninterruptible power supplies (UPSs), its turn-off incapability and the low frequency of operation (50–60 Hz) limited its application, it is alleged that the thyristor has reached saturation as far as its application is concerned [135]. In Figure 11, the schematic of a GTO is presented, it is seen to be very similar to that of the thyristor except that it can be turned-off at the gate.

Again, the GTO just like the SCR is not without its unique challenges such as poor turn-off current gain and the second-breakdown problem it exhibits at turn-off. It also has a limited operating frequency of less than 5 kHz and the gate drive design is quite complex due to the need for a large reverse gate current to turn it off [136].

**Figure 12:**
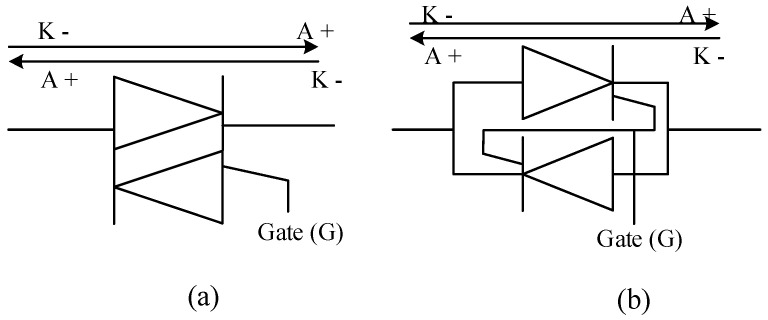
Schematic symbol of (**a**) a triode ac switch (Triac) and (**b**) the detailed depiction of a Triac’s formation from two SCRs [138].

Another interesting power electronic device in the thyristor family is the triode AC switch also known as the triac. The schematic of a triac is presented in Figure 12a. It can be observed that a triac is simply the arrangement of two thyristors in reverse-parallel configuration in a single chip. This is more clearly depicted in Figure 12b, the gates of the two SCRs are tied together and controlled from one source [137].

Although the triac is more economical to implement than just taking two thyristors and arranging them as described in Figure 12b, its current sensitivity is worse thus having a longer turn-off time due to the minority carrier storage effects. Its frequency of operation is similar to that of a thyristor at 50–60Hz. The triac is used in solid state relays, light dimming, heating control, and some home appliances [138].

**Figure 13:**
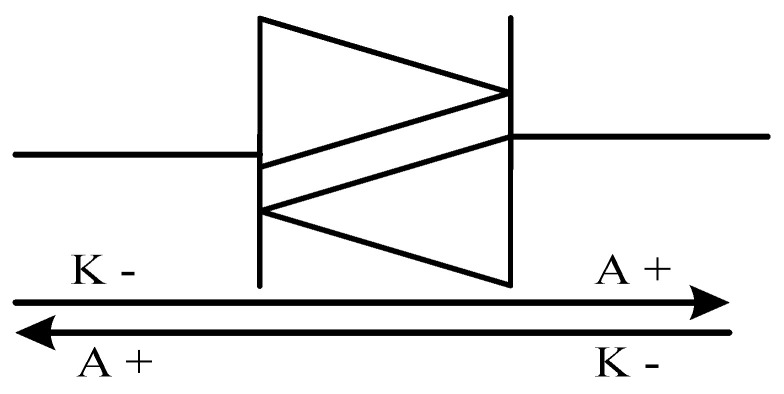
Schematic symbol of a diode AC switch (Diac) [139].

The story of the thyristor family will be incomplete without mentioning the diode AC switch otherwise known as the diac. The schematic symbol of a diac is presented in Figure 13. It is very similar to a triac except that a diac has no gate. Just like a triac, a diac has arbitrary terminal designations since it is a bilateral device. The diac is reported to find its application chiefly in switching or triggering the triac [139].

**Figure 14:**
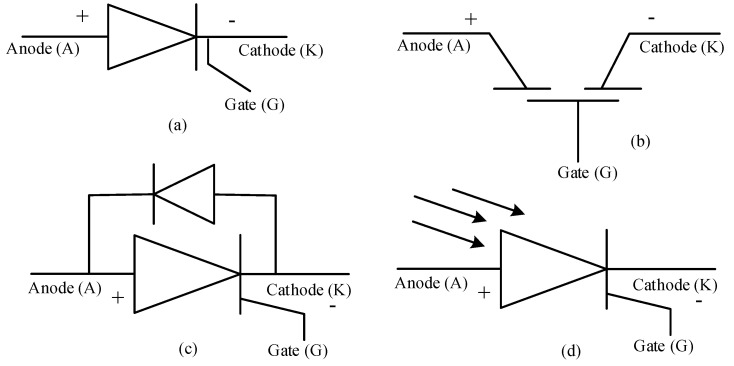
Schematic symbol of (**a**) a SITH, (**b**) an MCT, (**c**) an RCT and (**d**) an LASCR [140].

Other prominent members of the thyristor family include: the static induction thyristors (SITHs), MOS-controlled thyristor (MCT), reverse conducting thyristor (RCT), and the light activated-SCR (LASCR). Their respective schematic symbols are presented in Figure 14 [140].

The SITHs were introduced around 1988 and are turned-off in a similar way to the GTO, the downside of the SITH when compared with the GTO is that it has a higher conduction drop [141,142]. It is used in induction heating applications and static var compensators. MCTs were also introduced around the same time as SITHs and also has a high gain at turn-off just like the GTO. The MCT is continuously being improved and could pose a potential threat to the IGBT as it has a smaller voltage drop and can operate in higher temperatures [137,142]. RCTs are basically achieved by the inclusion of an anti-parallel body diode across an SCR in order to achieve current flow in the opposite direction [143]. The LASCR, as their name implies are light activated and is utilized in high voltage and current systems to achieve total electrical isolation between the control and the power sections of the system [122].

## 4. Summary

To draw the curtain on the overview of these modern power electronic switches, it is important to compare these switches, some of the most prominent of them being the GTOs, BJTs, SCRs, MOSFETs, and IGBTs. Comparing the GTOs to SCRs, the GTO triumph the SCR due to their faster turn-offs, their use results in increased converter efficiency, there is an elimination of the need for commutating components, there is also a reduction in electromagnetic and acoustic noise as a result of the elimination of chokes. GTOs also have a higher on-state gain, ratio of peak surge to average current and higher voltage blocking capabilities when compared with BJTs. However, GTOs require snubbers to operate normally and snubbers for high power applications are quite expensive. The high on-state voltage drop in GTOs coupled with the high gate current requirement and the high losses in its gate drive are quite a number of demerits of the GTO. The MCT, seems to be an improved GTO seeing it has a lower forward voltage drop, a high current density and an improved gate technology, hence making it easy to control.

On the side of the transistors, the IGBT which combines the advantages of the MOSFET and BJT seems to be the champion. It has a simple gate drive circuit and requires small amount of energy at switching which is a result of its high gate impedance. Although it has a lower switching speed when compared to the MOSFET, its current and voltage capability is higher than that of the BJT and MOSFET especially when compared 1 to 1. The MOSFET has the highest switching speeds and has the potential to be the most effective parallel switch. The SCR has the highest current and voltage ratings and over current protection can be achieved with a fuse. The IGBT as well as the MOSFET have some of the easiest gate drive features among the switches.

Summarily, the different power electronic switching devices although compete on several levels, still possess unique characteristics that make them suitable for very uniquely specific applications. Having said that, the IGBT and the MCT show a lot of promise when looking at the transistor and the thyristor families of power electronic devices respectively. One major difference that can be quickly observed is that devices with relatively large power capabilities have comparatively low frequencies of operation when compared to the devices with significantly lower power capabilities. More detail on the specific characteristics of each of the power electronic switches is presented in power electronics textbooks and many other relevant materials such as [27,115,144,145,146].

There is still room for continuous improvements and advancements in power electronic switches as their applications can be continuously improved as the switches get better by the years. In Table 1, the power electronic switches are compared based on their classification and there is an empty cell shaded grey. This shaded empty cell represents an opportunity for growth and improvements. It is the cell that represents the most ideal switch, a switch that supports the bidirectional flow of current and is fully controllable in both directions.

**Figure 15:**
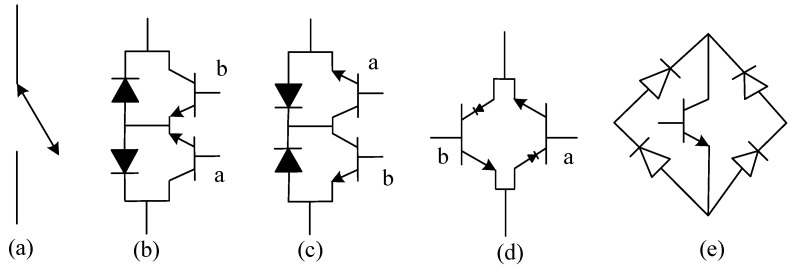
Schematic implementation of (**a**) the ideal switch and the different possible implementation of the four quadrant switch with (**b**) two IGBTs each with an antiparallel diode connected anti-serially in common emitter configuration (**c**) two IGBTs each with an antiparallel diode connected anti-serially in common collector configuration (**d**) two reverse biasing IGBTs connected in antiparallel configuration and (**e**) diode bridge configuration with one IGBT [70].

In order to fill the gap highlighted in grey on Table 1, the modular configuration of switches in a manner in which they have the capacity to achieve bidirectional flow of current while still being fully controllable in both directions shows a strong potential. This modular configuration is often referred to as the four-quadrant switch, this is because the switch is capable of operating, in all four quadrants of power electronics switch operation, that is their ideal *V*-*I* characteristics. The implementation of the four quadrant switch is presented in Figure 15 [27,70,146]. As seen in Figure 15a,b the four-quadrant operation is achieved by connecting two discrete switches (either IGBT or MOSFET) with anti-parallel body diodes connected in anti-series configuration, thereby achieving common emitter and common collector configuration. In Figure 15b, two reverse blocking switches are connected in anti-parallel to achieve four quadrant operation. The use of reverse blocking switches have become a subject or research as they now bring a possibility of reducing the two diodes that would have been used in Figure 15a,b [147]. The configuration in 15d uses a diode bridge and a switch, this might be functional in some matrix converters but increases losses and might not be applicable in multi-level inverters [148].

**Figure 16:**
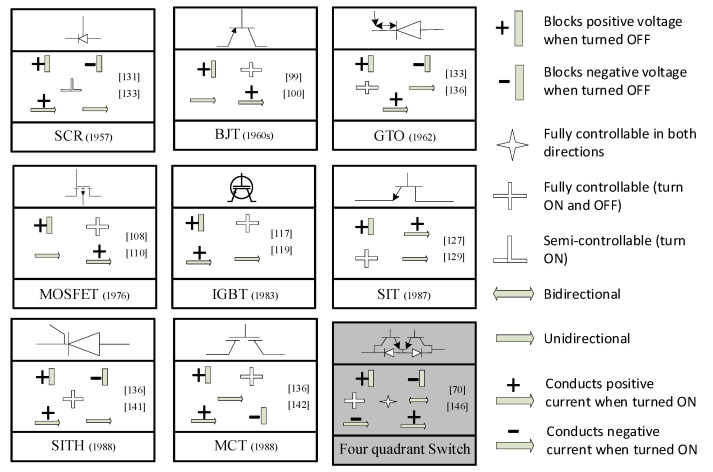
Comparison of some of the prominent power electronic switches for their controllability and ideal *V*-*I* characteristics.

Also, in Figure 16, a summary-comparison of the ideal *V-I* and other characteristics of some of the most prominent power electronic switches is presented, it is seen that the ideal switch has some of the most desirable characteristics when it comes to bidirectional current flow and voltage blocking characteristics among many others. Although a couple of research has gone into investigating the four-quadrant switch, the dearth of possible applications aside in matrix converters might have limited its widespread adoption, however, the fully controllable bidirectional switch presents a lot of potential. Also, the difficulty that may arise in the miniaturization and combination of the four-quadrant switch into a single chip might have also slowed its manufacturing and adoption.

However, thanks to the benefits of wide band gap technology, some of these challenges will soon be overcome if more research goes into solving the respective problems. Recently, the field of power electronic devices has awakened to the wide band gap technology and this has resulted in the birthing of the high electron mobility transistors (HEMTs) [149,150,151,152,153,154,155,156,157,158,159,160,161,162,163,164,165,166,167,168,169,170]. These transistors, mostly MOSFETs and IGBTs, are made from materials such as silicon carbide (SiC), gallium nitride (GaN), indium phosphide (InP), aluminum gallium nitride (AlGaN), etc. to list just a few, have continued to show a lot of promise as improvements are being made faster and faster as the different researches surface [171,172,173,174,175,176,177,178,179,180,181,182,183]. Taking advantage of this technology, manufacturers of power electronic switches can now begin to look into making the four quadrant switches.

There is also need on the front of rese arch to examine the different possible configuration of the four quadrant switches to establish the most suitable configuration for a number of applications based on the achievable efficiencies, power densities and many other figures of merit. More research needs to also go into the possible adoption of four quadrant switches in many applications, especially in the area of multiple input converters, which require a fully controllable bidirectional flow of power in order to replace whatever the current substitute might be. Furthermore, there are currently few options of four quadrant switches on the market such as the SKMxxxGM12T4 from Semikron which is only limited to 20 kHz switching frequency [184]. Also the use of reverse blocking IGBTS also needs more work both in research and industry since only few of these are currently on the market prominently only the FGW85N60RB from Fuji Electric [185]. In considering the possibility for monolithic four quadrant switches, lessons can be learnt from the new GaNFET from Texas Instruments, the LMG341xRxxx series [186]. This family of devices features an integrated gate driver with either cycle by cycle or latched over current protection and a low on state resistance as low as 50 mΩ. This places more emphasis that with WBG technology much more can be done in power electronic switches.

Selecting semiconductor devices can be a painstaking task with the plethora of properties and characteristics available for different devices and materials. This task is made easier with the many different figures of merit (FOM) that have been proposed in literature [28]. Some of the most prominent have been presented on Table 2. These are classified into two major categories, FOMs for comparing devices like IGBTs, MOSFETs etc. and those for materials. The different applications for these FOMs are described on the table as well. One FOM that stands out is the power density (PDFOM) proposed by [187], this FOM can be used in selecting devices when designing power converters. The PDFOM could really become a game changer for selecting and designing power converters, since they are key drivers in renewable energy power systems. It is, however, important to carry out further studies into the application of these FOMs from an end user point of view to ensure they accurately predict the performance characteristic they are proposed to define. Furthermore, it is important to note that the BHFFOM and NHFOM should be used together and not exclusively for comparing devices to be used in high frequency applications. This as they consider the switching loses due to the input and output capacitance of the switch, respectively. Since the field of semiconductor devices and materials is rapidly growing it is difficult to compare based on cost for future outlook. It is expected that cost will go down as the technologies mature and adoption increases.

Also, the relationship between the losses in a semiconductor device due to its material make up is highlighted in the BFOM and the HMFOM. Both FOMs are proposed for evaluating the conduction losses and the power losses, respectively. It is seen that the main parameters that are most responsible for the loss performance of the semiconductor device due to its material are the critical electric field, the carrier mobility, and the dielectric constant of the semiconductor material from which the device is made. The higher these values, then the respective device made from such material will have lower power losses. When the material properties presented in Section 6 are examined, it is seen that materials with wide bandgap will generally result in having lower losses as their critical electric field and carrier mobility values are high. The materials in the ultrawide bandgap will have some of the highest FOMs and so will result in switches with lower losses when the technology matures further and goes into commercial manufacturing.

## 5. Reliability of Power Electronic Switches

With the widespread application of power electronic switches, especially in renewable energy systems, comes an increased concern for their reliability and failure rates. Studies have reported that power converters accounted for 38%, 37%, and 13% of unplanned maintenance in variable ac speed drives, photovoltaic and wind power systems respectively [192]. Also, semiconductor devices have been observed to be the most fragile component of power electronic systems as the device and package related failures account for up to 35% of the failures in the respective systems [193]. This highlights the importance of reliability in the application of power electronic switches. The main failures in semiconductor devices are caused by mechanical and thermal stresses [194], therefore temperature and power cycling have been the most common approaches utilized in evaluating the reliability of devices. The process of temperature and thermal cycling is described in [195,196].

It is also beneficial to predict the mean time to failure (MTTF) and hence the reliability of semiconductor devices as this will lead to a good understanding of the mean time between failure (MTBF) of systems they are utilized in. To achieve this, the military handbook on reliability prediction of electronic equipment, (MIL-HDBK-217F) [197] is most commonly used as a guideline [198]. Table 3 defines the formulas for calculating the different failure rates, $\lambda_{p}$, for different semiconductor devices as defined by MIL-HDBK-217F. Where $\lambda_{b}$ is the base failure rate due to the influence of electrical and temperature stresses on the device, $\pi_{E}$ includes the effect of environmental factors such as: if the device is used in space, ground, airborne etc., $\pi_{T}$ represents the factor of temperature, particularly the junction temperature, $\pi_{S}$ factors in the electrical stress factor, (that is the relationship between the rated voltage and applied voltage), $\pi_{Q}$ factors in the quality of the device packaging, $\pi_{C}$ factors in the contact construction, $\pi_{R}$ factors in the power rating of the device, $\pi_{A}$ factors in the application of the device and $\pi_{M}$ includes the matching network factor. From table x, in predicting the reliability, the factors of temperature, quality and environment of the device is applied in all cases.

Another resource often used in predicting reliability of semiconductor devices is the IEC-TR-62380 is presented as Equation (1). Where $\pi_{U}$ is the use factor (whether temporary or permanent), $\lambda_{0}$ is the base failure rate of the semiconductor die, ${\left(\pi_{t}\right)}_{i}$ is the temperature factor of the device (relating to the junction temperature), $\tau_{i}$ is the working time ratio of the device for the corresponding junction temperature, $\tau_{on}$ is the total working time ratio of the device, $\tau_{off}$ time ratio of the device being in idle mode, ${\left(\pi_{n}\right)}_{i}$ is the influence factor from the annual cycles number of thermal variations applied on the package, $\Delta T_{i}$ is the amplitude variation due to the respective thermal variation, $\lambda_{B}$ is the base failure rate of the device package, $\pi_{I}$ is the factor relating to whether the device is used with a protection interface or not, and $\lambda_{EOS}$ is the electrical overstress of the device in the considered application.

(1) $$\lambda_{p}=\left[\underset{\lambda_{die}}{\underset{︸}{\left(\pi_{U}\times \lambda_{0}\right)\times \left\{\frac{\sum_{i=1}^{y}{\left(\pi_{t}\right)}_{i}\times \tau_{i}}{\tau_{on}+\tau_{off}}\right\}}}+\underset{\lambda_{package}}{\underset{︸}{\left\{\left[2.75\times 10^{-3}\times \sum_{i=1}^{z}{\left(\pi_{n}\right)}_{i}\times {\left(\Delta T_{i}\right)}^{0.68}\right]\times \lambda_{B}\right\}}}+\underset{\lambda_{overstress}}{\underset{︸}{\left\{\pi_{I}\times \lambda_{EOS}\right\}}}\right]\times 10^{-9}/hour$$

Although Equation (1) seems generic for all semiconductor devices, it is more complex and complicated than the proposal of the MIL-HDBK-217F as all the parameters are different for each specific device, depending on the package type, switching frequency, type of device and application. The IEC-TR-62380 also gives an indication of the failure rates of the different aspects of the device. The specific values of all the different variables can be found in the respective documents. Generally, the two different guidelines propose different methods of calculating the failure rate ($\lambda_{p}$) of each component. From this value of $\lambda_{p}$, the MTTF can be calculated as $MTTF=\lambda_{p}^{-1}$ and subsequently, the reliability (R) is calculated as $R=e^{-\lambda_{p}t}$. It is important to note that with wide bandgap technology, it is unclear if these prediction recommendations are valid. Therefore, the need for investigations to verify this, and to also compare both guidelines which more accurately predict the failure rates. This can be considered for the future of the white paper being developed by the IEEE for wide bandgap technology applications, the international technology roadmap for wide bandgap power semiconductors (ITRW) [199].

## 6. Future Trends

The future of power electronic switches is brighter with the introduction of wide bandgap technology. It is even, more promising considering the amount of research going into ultrawide bandgap semiconductor materials. Although, the technology of ultrawide bandgap semiconductors is still immature. It shows a lot of promise as the materials forming this class of semiconductors have bandgaps up to 6-times that of silicon. The properties of some of these materials is presented on Table 4. Semiconductors made from diamond, popular for being the hardest material, could likely be the most promising super device of the future. This due to their ultrawide bandgap, high thermal conductivity, electron, and hole mobility among other attractive features. The successes achieved in realizing single-crystal man-made substrates make diamond electronics even more promising [200]. AlGaN/AlN on the other hand while having similar attractive features of diamond, has a major challenge of available single-crystal substrates with good enough quality for epitaxial growth. Cubic-Boron-Nitride (c-BN) being next to diamond as the second hardest material also shows some promise in the future of power electronic switch manufacturing. This is if the challenges surrounding its synthesis are overcome [201]. β-Ga_2_-O_3_ is one of the newest entries in the ultrawide-bandgap class of semiconductors, its properties and availability makes it particularly attractive. Its main disadvantage is its poor thermal conductivity and a lack of success in p-type doping [202].

## 7. Conclusions

In conclusion, the power electronics devices field is still an open field with a lot of opportunities for improvements and advancements especially in the transistor families. With the emergence of the high electron mobility transistors (HEMTs), through the superhighway of wide band gap technology. Only little of what can be achieved has been done and there is no doubt that the HEMTs are the devices of the future.

One switch, the four-quadrant switch, which has seemingly been neglected by researchers and manufacturers, was discussed in this paper. This neglect has probably arisen due to a dearth of research into its possible applications, these needs to be looked into by future research as the four-quadrant switch holds a lot of promise since it is the closest switch to an ideal switch. With the advantages of wide band gap technology, there is little or no doubt that the opportunities of the four-quadrant switch can be exploited.

Finally, the figures of merit for comparing semiconductor devices and materials were presented. These FOMs help in objectively comparing different devices for specific applications as different applications require different unique characteristics and some devices are better at certain characteristics and worse for other characteristics. The PDFOM is useful for application in designing high density power converters and so should be taken into account when comparing power converter topologies for renewable energy applications.

## Figures and Tables

**Table 1:** Comparison of the Power Electronic Switches across their Controllability and Bidirectional Capability.

	Uncontrollable	Semi-Controllable	Fully Controllable
Unidirectional	Diode	Thyristor	BJT, MOSFET, GTO, SIT, SITH, IGBT, SITH and MCT
Bidirectional	Diac	Triac	

**Table 2:** Figure of Merits (FOM) for Comparing Semiconductor Devices and Materials [28,187,188,189].

FOM		Equation	Application
Materials	JFOM	$\frac{E_{c}v_{s}}{2\pi }$	Definition of cut-off frequency and maximum applicable voltage. Useful for choosing devices in signal amplifications.
	KFOM	$\lambda \frac{cv_{s}}{4\pi \varepsilon }$	Time delay of transistor action, essentially an indication of the switching speed limits.
	BFOM	$\varepsilon \mu E_{c}^{3}$	Comparison to highlight conduction losses.
	HMFOM	$E_{c}\sqrt{\mu }$	Comparing power loss in different materials.
	HCAFOM	$\varepsilon E_{c}^{2}\sqrt{\mu }$	Chip area comparison of materials.
	HTFOM	$\frac{\lambda }{\varepsilon E_{c}}$	Comparison of different materials to highlight thermal performances.
Devices	BHFFOM	$\frac{1}{R_{on}C_{in}}$	Switching loss due to input capacitance and Conduction loss comparison for high frequency application.
	NHFFOM	$\frac{1}{R_{on}C_{oss}}$	Switching loss due to output capacitance and Conduction loss comparison for high frequency application.
	FOM_(O)_	$\frac{V_{c}I_{c}}{V_{on}t_{off}S}$	Comparison considering power rating, power loss and chip area of the device. Does not consider structural parameters of the device.
	FOM_(S)_	$\frac{V_{c}I_{c}}{V_{on}t_{off}SR_{th}L_{s}}$	Comparison considering power rating, power loss, chip area and thermal resistance of the device. Considers device structural parameters.
	PDFOM	$\frac{1}{A_{pack}R_{th}\sqrt{R_{on}Q_{gd}}}$	Comparison of devices considering packaging, thermal properties and loss for application in converters.
	HDFOM	$\sqrt{R_{on}Q_{gd}}$	Comparing power loss in different devices ignoring the loss in the gate switching circuit.

$E_{c}$ = Critical electric field, $v_{s}$ = Carrier saturation drift velocity, λ = Thermal conductivity, c = velocity of light in free space, ε = Dielectric constant of the semiconductor, μ = Carrier mobility, $C_{in}$ = Input capacitance of the device, $C_{oss}$ = Output capacitance of the device, $V_{c}$ = Device rated voltage, $I_{c}$ = Device rated current, $V_{on}$ = On-state voltage of the device, $t_{off}$ = Turn-off time of the device, S = Die area of the device, $L_{s}$ = Stray inductance of the device, $A_{pack}$ = Device package area, $R_{th}$ = Thermal resistance of the device,$R_{on}$ = On-state resistance of the device, and $Q_{gd}$ = miller charge. J—Johnson, K—Keyes, B—Baliga, HM—Huang material, HCA—Huang Chip Area, HT—Huang Thermal, BHF—Baliga High Frequency, NHF—New High Frequency, PD—Power Density, HD—Huang Device, _O_—FOM proposed by Ohmi and Takeuchi [190], and _S_—FOM proposed by Shigekane et al. [191].

**Table 3:** Definition of failure rates for different devices according to the MIL-HDBK-217F [197].

Device	$\mathrm{Failure}\;\mathrm{Rate}\lambda_{p}\;(\mathrm{Failures}/10^{6}\;h)$
Diode (Low Frequency)	$\lambda_{b}\pi_{T}\pi_{S}\pi_{C}\pi_{Q}\pi_{E}$
Diode (High Frequency)	$\lambda_{b}\pi_{T}\pi_{A}\pi_{R}\pi_{Q}\pi_{E}$
Transistors (Low Frequency, Bipolar)	$\lambda_{b}\pi_{T}\pi_{A}\pi_{R}\pi_{S}\pi_{Q}\pi_{E}$
Transistors (Low Frequency, Si FET)	$\lambda_{b}\pi_{T}\pi_{A}\pi_{Q}\pi_{E}$
Transistors (Unijunction)	$\lambda_{b}\pi_{T}\pi_{Q}\pi_{E}$
Transistors (Low Power, High Frequency, Bipolar)	$\lambda_{b}\pi_{T}\pi_{R}\pi_{S}\pi_{Q}\pi_{E}$
Transistors (High Power, High Frequency)	$\lambda_{b}\pi_{T}\pi_{A}\pi_{M}\pi_{Q}\pi_{E}$
Transistors (High Frequency, GaAs FET)	$\lambda_{b}\pi_{T}\pi_{A}\pi_{M}\pi_{Q}\pi_{E}$
Transistors (Low Power, High Frequency, Si FET)	$\lambda_{b}\pi_{T}\pi_{Q}\pi_{E}$
Thyristors, SCRs and Triacs	$\lambda_{b}\pi_{T}\pi_{R}\pi_{S}\pi_{Q}\pi_{E}$

**Table 4:** Properties and Electrical Characteristics of Semiconductor Materials [20,148,189,203,204,205,206,207,208,209,210,211].

Material Property		Wide Band Gap							Ultra-Wide Band Gap			
	Si	SiC	3C-SiC	4H-SiC	6H-SiC	GaN	GaAs	GaP	Diamond	AlGaN/AlN	β-Ga_2_-O_3_	c-BN
Bandgap, E_g_ (eV)	1.12	3.2	2.3	3.26	2.9	3.4	1.4	2.26	5.5	6.0	4.9	6.4
Thermal Conductivity (W/m.K)	253	145	500	500	500	253	52	110	2290–3450	253–319	11–27	940–2145
Electron Mobility, µ_n_ (cm^2^/V.s)	1400	700	1000	800	415	2000	8500	250	2000	426	150	825
Hole Mobility, µ_p_ (cm^2^/V.s)	450	100	45	115	101	350	400	150	1800	30	-	500
Electron Saturation Velocity, V_nsat_ (10^6^ cm/s)	10	20	25	22	19	14	44	20	23/13 (e^−^/h^+^)	13	11	-
Critical Electric Field, E_c_ (MV/cm)	0.3	3.2	2	3.18	2.4	4.9	0.4	1	13.0	15.4	10.3	17.5
Relative permittivity	11.9	10	9.6	9.7	9.7	10.4	12.9	11.1	5.7	9.76	10.0	7.1
